# Precursor-based surface modification of cathodes using Ta and W for sulfide-based all-solid-state batteries

**DOI:** 10.1038/s41598-020-67493-6

**Published:** 2020-06-29

**Authors:** Chung Bum Lim, Yong Joon Park

**Affiliations:** Department of Advanced Materials Engineering, Graduate School Kyonggi University, 154-42, Gwanggyosan-Ro, Yeongtong-Gu, Suwon-Si, Gyeonggi-Do 16227 Republic of Korea

**Keywords:** Batteries, Materials for energy and catalysis

## Abstract

Sulfide ionic conductors are promising candidates as solid electrolytes for all-solid-state batteries due to their high conductivity. However, interfacial instability between cathodes and sulfide electrolytes still remains a challenge because sulfides are highly reactive. To suppress undesirable side reactions at the cathode/sulfide electrolyte interface, the surface of the cathode has been modified using stable coating materials. Herein, a precursor based (PB) surface modification using Ta and W is introduced as an effective approach for the formation of a suitable cathode coating layer. Through heat-treatment of the PB surface modification, the source materials (Ta or W) coated on the precursors diffused into the cathode and acted as a dopant. Formation of the surface coating layer was confirmed by X-ray photoelectron spectroscopy (XPS) depth profiles and scanning transmission electron microscopy (STEM) images. The PB surface modified electrodes showed higher capacity, improved rate capability and enhanced cyclic performance compared to those of the pristine electrode. The impedance value of the cells dominantly decreased after cycling due to the modification effect. Moreover, considering the XPS analysis, undesirable reaction products that formed upon cycling were reduced by PB surface modification. These results indicate that PB surface modification using Ta and W effectively suppresses undesirable side reactions and stabilizes the cathode/sulfide electrolyte interface, which is a synergic effect of the doping and coating attributed to Ta and W.

## Introduction

Recently, all-solid-state lithium batteries (ASSLBs) employing inorganic solid electrolytes have attracted sizable attention because of their many advantages. The main benefit of ASSLBs is their superior safety compared to commercial lithium ion batteries (LIB), which stems from the adoption of non-flammable solid electrolytes instead of flammable liquid electrolytes^1–7^. A wide operation temperature is also an important potential advantage of ASSLBs. Inorganic solid electrolytes are more stable at low and high temperatures (for example, -50~200 °C) in which liquid electrolytes cannot sustain their properties^8–10^. In addition, the low activation energies of solid electrolytes could reduce variations in ionic conductivity that occur with temperature^8^. These facts support their use for reliable battery operation in a wide temperature range. Their higher volumetric and gravimetric energy densities than commercial LIB have been suggested as other expected advantages because ASSLBs adopt the bipolar staking of the anode of one cell and can easily use lithium anodes^2,8,11,12^.

Despite these great potential benefits, ASSLBs face many challenges, such as the low ionic conductivity of solid electrolytes and high interfacial resistance between electrolytes and electrodes. The development of a superionic conductor for solid electrolytes remains the most important mission for the realization of ASSLBs. For several decades, many ionic conductors have been explored as candidate materials^13–17^. These endeavours resulted in a dramatic increase in the ionic conductivity of solid electrolytes. In particular, several state-of-the-art sulfide electrolytes, such as Li_10_GeP_2_S_12_ (LGPS) and Li_9.54_Si_1.74_P_1.44_S_11.7_Cl_0.3_ (LSiPSCl), have even demonstrated superior conductivity to organic liquid electrolytes^11,14^. Furthermore, good ductility and high sulfide elasticity enables the formation of interfacial connections between solid electrolytes and electrodes through a mechanical process without using high temperature sintering^7–11^. Thus, ASSLBs based on sulfide solid electrolytes have been considered as one of the most commercially viable next generation battery systems. However, the issue of high interfacial resistance remains a serious challenge for sulfide-based ASSLBs. In particular, the unstable interface between sulfides and oxide cathodes results in the degradation of the electrochemical performance of ASSLBs.

So far, the sulfide electrolyte/cathode interface issues can be classified as follows. The first issue is the side reactions between sulfides and oxide cathodes, which produces undesirable interface layers with low ionic conductivity^18–20^. The second is the formation of a space charge layer due to the decomposition of sulfide electrolytes at the sulfide electrolyte/cathode interface^19,21,22^, which retards the migration of lithium ions during the charging/discharging process. The third is unstable mechanical contact between the cathodes and electrolytes due to the expansion/contraction of the cathode during cycling^18^. The high interfacial resistance of ASSLB based on the sulfide electrolyte is mainly attributed to these factors, so controlling them is a key issue for the commercialization of ASSLBs.

The surface modification of cathodes has been suggested as an effective approach to reducing side reactions and suppressing the formation of the space charge layer. For decades, surface coating has contributed to reducing the reaction between the HF in the liquid electrolyte and the cathode surface to produce a stable LIB system^23–27^. However, the coating layer on the surface of the cathodes in sulfide-based ASSLBs must play a different role, that is, the suppression of the side-reaction with sulfides. Moreover, the coating material should have good ionic conductivity because solid electrolytes do not penetrate into cathodes like liquid electrolytes. Several oxides, such as Li_2_ZrO_3_^28–30^, Li_2_MoO_4_^31^, LiInO_2_^32^, Li_2_Ti_5_O_12_^33^, and LiNbO_3_^21,34^, have been reported as coating materials that meet these conditions. However, the surface modification of ASSLB is still in its initial stages compared to LIBs. In particular, research on coating materials and coating methods suitable for ASSLBs is necessary.

We considered a more effective process through which to form a uniform surface layer and obtain a superior surface modification effect. The method used is mainly to synthesize cathodes. The as-synthesized cathodes are then surface modified using coating solutions and heat treatment. This post-coating process is suitable for coating oxides composed of one cation, such as Al_2_O_3_ or ZrO_2_, and it has been used for coating materials for LIB systems. However, the coating materials for ASSLBs need to consist of two cations (such as Li and other cations) because they should have the ionic conductivity of Li^+^. For the formation of these oxides, the calcination temperature should be increased to 700–900 °C, which may result in the degradation of the electrochemical performance of the cathode because Li^+^ will dissolve out of the host phase at high temperatures^35,36^. To solve this problem, precursor-based (PB) surface modification is proposed as a suitable method for improving ASSLBs in this study. The solution-based coating process was applied to the precursors (Ni_0.6_Co_0.2_Mn_0.2_ (OH)_2_), instead of the as-synthesized cathode. Next, the coated precursors were lithiated through calcination with LiOH·H_2_O at a high temperature (800 °C) to form LiNi_0.6_Co_0.2_Mn_0.2_O_2_ and a coating layer. During this process, the lithium and other cation ions used for precursor-coating react sufficiently to form a stable coating layer, where the cations can also migrate into the parent-cathode, acting as a dopant. Furthermore, calcination at a high temperature may lead to a thin and homogeneous surface modification compared to the post-coating process. Ta and W were selected as cation ions for the surface modification of the cathodes because they can form stable lithium ion conductors (LiTaO_3_, LiWO_3_ or Li_2_WO_4_) and have been used as stable coating materials^37,38^. We also considered that Ta and W are effective doping materials for the stabilizing phase of high Ni cathodes and for enhancing their electrochemical performance^39,40,41^. To analyse the effect of PB surface modification using Ta and W, the electrochemical performance of modified LiNi_0.6_Co_0.2_Mn_0.2_O_2_ cathodes was characterized using sulfide electrolytes. Scanning electron microscopy (SEM), scanning transmission electron microscopy (STEM), energy dispersive X-ray spectroscopy (EDS), X-ray diffraction (XRD), and X-ray photoelectron spectroscopy (XPS) were performed to elucidate the effects of PB surface modification.

## Characterization of surface modified powders

**Figure 1:**
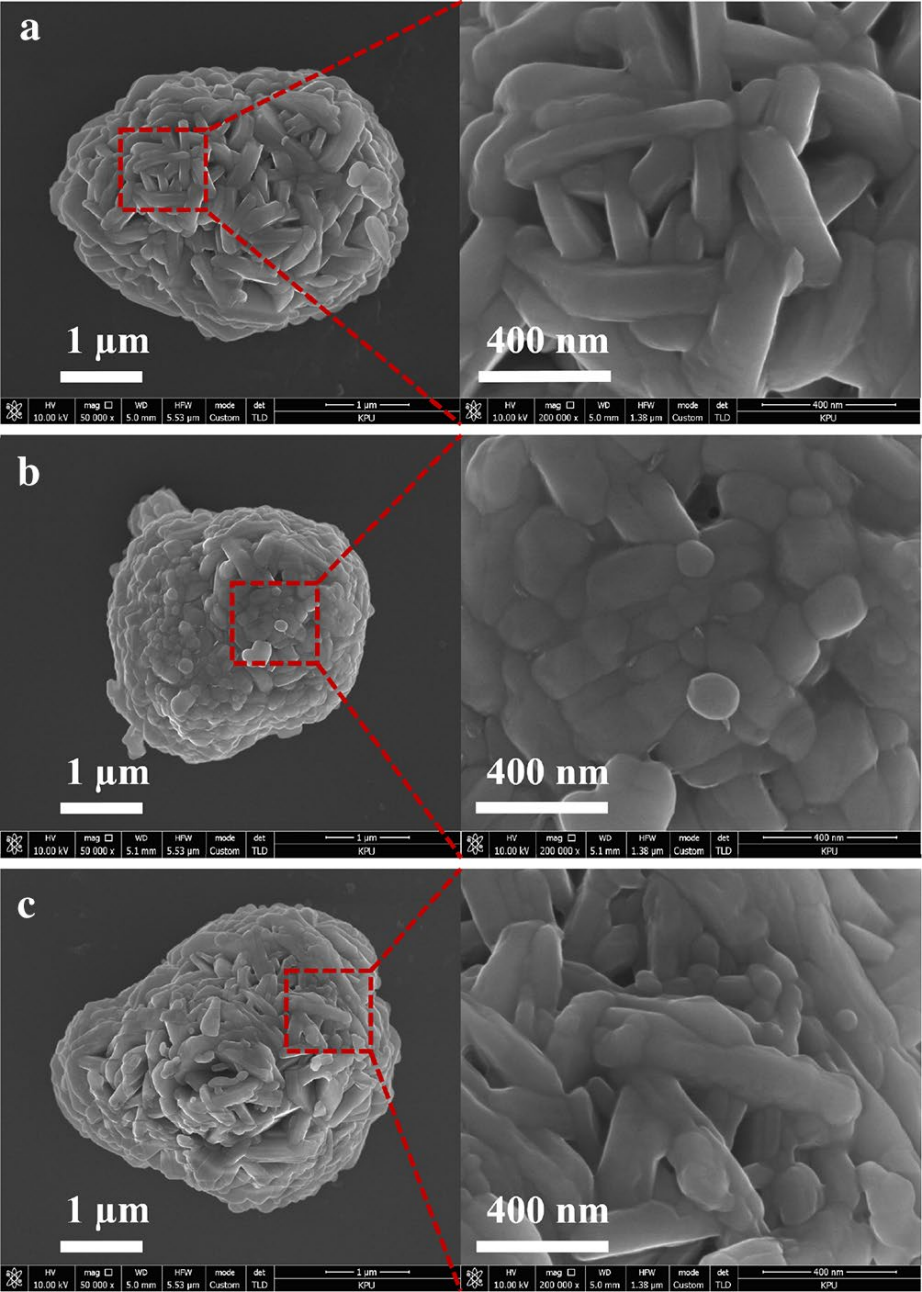
SEM images of the pristine and PB surface modified LiNi_0.6_Co_0.2_Mn_0.2_O_2_ powders: (**a**) pristine, (**b**) surface modified using Ta, and (**c**) surface modified using W.

Figure 1 shows the surface morphologies of the pristine and PB surface modified LiNi_0.6_Co_0.2_Mn_0.2_O_2_ powders as observed by SEM. The powders consisted of agglomerates of nano-sized granules. As shown in Fig. 1, the surface morphologies of the powders were not critically changed after surface modification. Compared to the pristine powder, the surface of the PB surface-modified powder seemed to be covered with a film-like layer; however, it was not clear in appearance in SEM images. Considering the fact that the surface coated samples prepared by the post-coating process were generally covered with nano-sized coating particles^31,32^, the samples prepared through PB surface modification had very smooth surface morphologies. The heating procedure for PB surface modification requires a relatively longer time and higher temperature than that of the general post-coating process. As a result, a significant portion of the source materials coated on the surface of the precursors may penetrate inside the cathode due to the ample diffusion time and high temperature, which creates a doping effect. In contrast, the surface coating layer cannot be clearly distinguished from the parent-cathodes because the surface layer is either very thin or forms a kind of concentration gradient.

**Figure 2:**
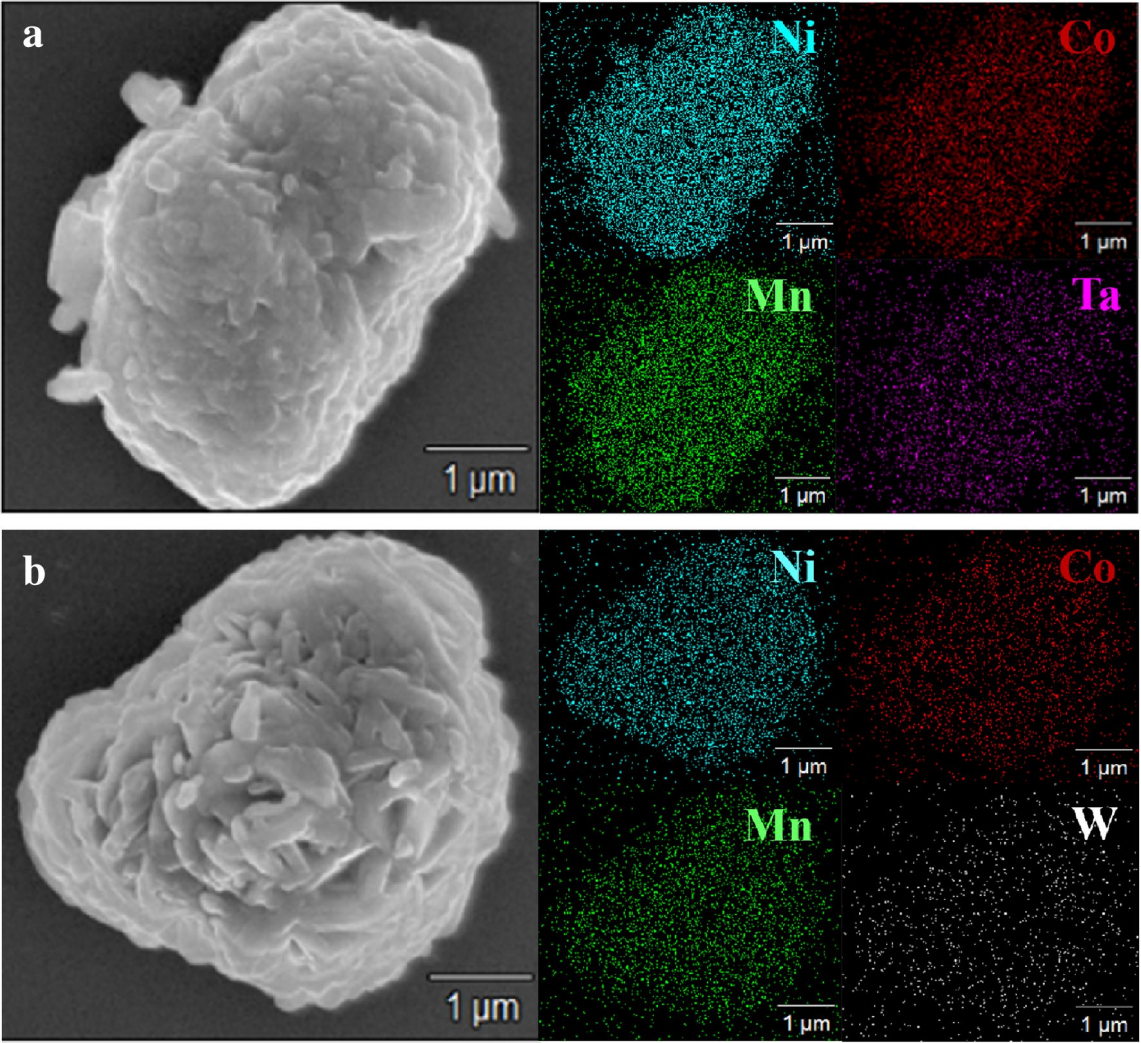
SEM and elemental mapping images of the PB surface modified LiNi_0.6_Co_0.2_Mn_0.2_O_2_ powders: (**a**) surface modified using Ta and (**b**) surface modified using W.

**Figure 3:**
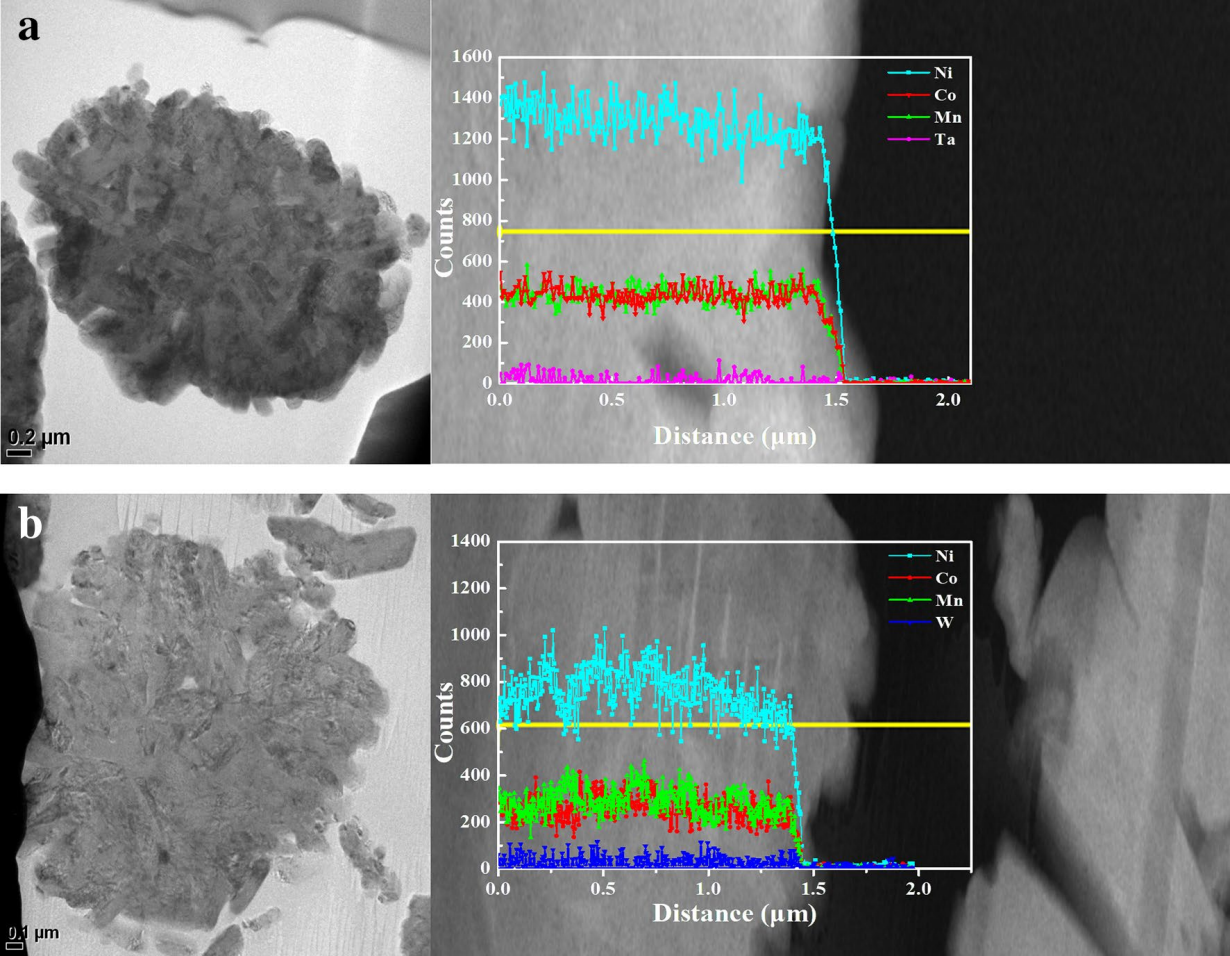
Cross-sectional HAADF-STEM image (left) and EDS line profiles (right) for Ni, Co, Mn, Ta and W. (**a**) PB surface modified using Ta and (**b**) PB surface modified using W.

To check the distribution of source ions (Ta and W), the surfaces and insides of the cathode powders were analyzed using SEM–EDS and cross-sectional STEM-EDS. Figure 2 presents the SEM and element mapping images of the PB surface modified powder using Ta and W sources. The Ta and W, as well as the Ni, Co, and Mn, were uniformly distributed on the surface of the PB surface modified powder, indicating that the source ions (Ta and W) were homogeneously dispersed on the surface. The diffusion of the source ions inside the cathode was observed using cross-sectional STEM images and EDS line profiles, as shown in Fig. 3. In the EDS line profiles of the cross-sectional STEM images, the source ions (Ta and W) were detected inside of the cathodes, indicating that those were diffused into the cathode. Although the sensitivity of EDS analysis was not enough for accurate quantitative measurements of Ta and W, it seemed that a considerable amount of source ions were distributed inside the cathode. Considering these results, the PB surface modified samples are expected to show a doping effect on the source ions.

**Figure 4:**
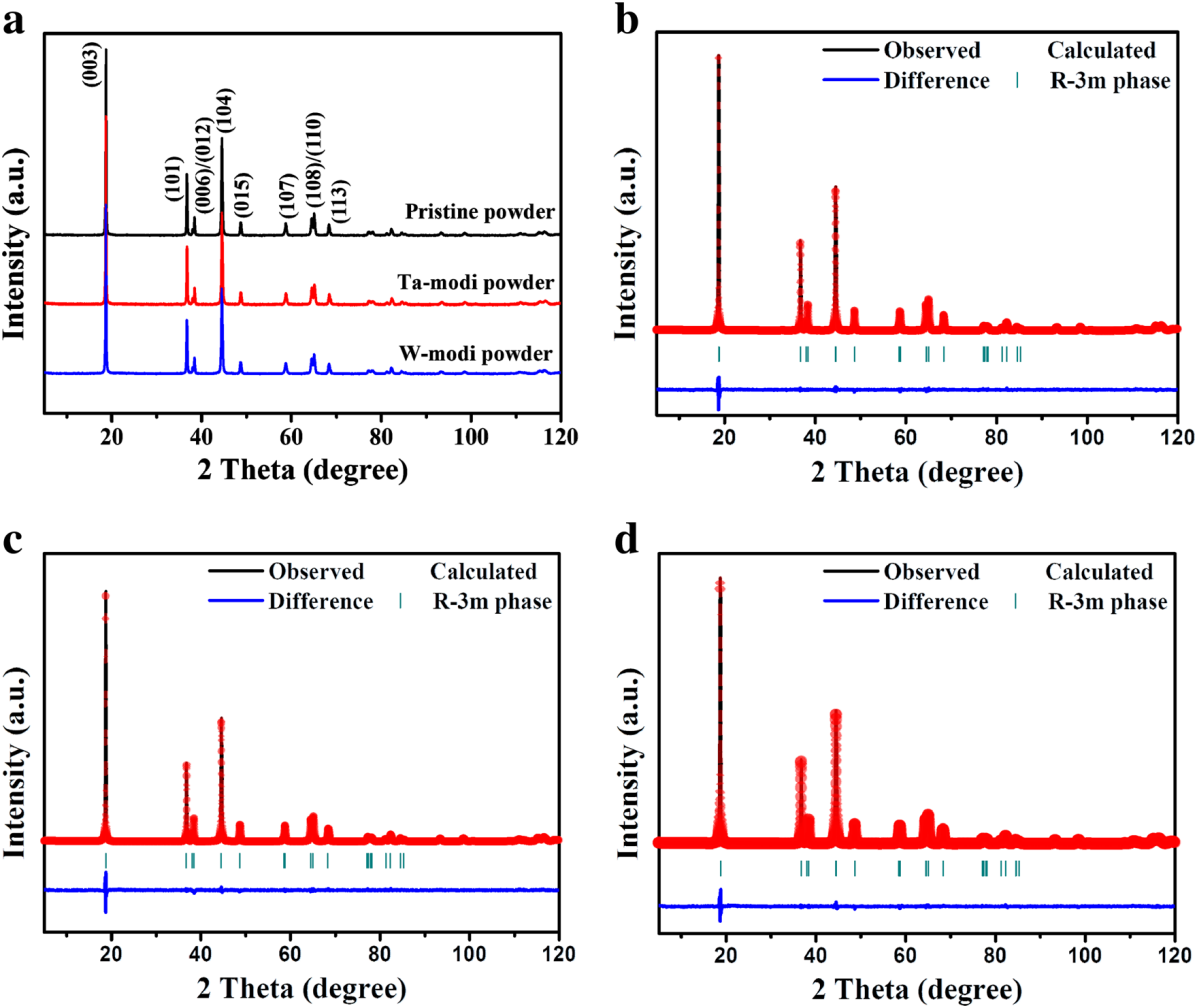
XRD patterns of pristine and PB surface modified powders. (**a**) XRD patterns of powder, and Rietveld refinements of the (**b**) pristine, (**c**) surface modified using Ta, and (**d**) surface modified using W.

The doping of Ta and W may affect the structure of the cathode. The crystal structure of the pristine and PB surface modified LiNi_0.6_Co_0.2_Mn_0.2_O_2_ powders were analyzed by XRD. As shown in Fig. 4a, all diffraction peaks of the samples can be well identified as an α-NaFeO_2_ structure (space group *R*$\overline{3}$*m*), which implies that the PB surface modification does not critically change the crystal structure. However, the positions of the diffraction peaks, such as (003), (006), (012), (108), and (110), were somewhat shifted by the modification (especially when using Ta), as shown in Supplementary Fig. S1. For more detailed characterization of the structure, the Rietveld refinement was used. Figure 4b–d presents the observed and calculated XRD patterns using the Rietveld refinement. The cell parameters derived from the Rietveld refinement and the I(003)/I(104) values of the samples are summarized in Table 1. Lattice parameters **a** and **c** of the pristine powder were 2.8647 Å and 14.206 Å, respectively. Those of the PB surface modified powder using Ta showed somewhat decreased **a** (2.8610 Å) and **c** (14.202 Å) values. The unit cell volume (**V**) also decreased, but the **c/a** ratio increased a little bit from the modification. These results imply that some Ta ions were incorporated into the LiNi_0.6_Co_0.2_Mn_0.2_O_2_ structure. Considering the ionic radius of transition metals such as Ni^2+^ (0.69 Å), Co^3+^ (0.545 Å), and Mn^4+^ (0.53 Å) in the LiNi_0.6_Co_0.2_Mn_0.2_O_2_, it is unlikely that Ta^5+^ (ionic radius = 0.64 Å) can substitute for the transition metals. Instead, it is reasonable that Ta^5+^ would have migrated to the Li sites because the ionic radius of Ta^5+^ is smaller than that of Li^+^ (0.76 Å), which can explain the decreased lattice parameters (**a** and **c**) and unit cell volume (**V**) from PB surface modification using Ta. A previous report has also shown that Ta^5+^ is located in the Li sites when it used as a cathode dopant^41^. The Ta^5+^ in the Li sites may act as a pillar during the movement of lithium ions and suppress the oxygen atom repulsion, which results in the enhancement of the structural stability of the LiNi_0.6_Co_0.2_Mn_0.2_O_2_ cathode. Moreover, it is notable that the I(003)/I(104) values significantly increased through PB surface modification using Ta. I(003)/I(104) gives information about the degree of cation mixing. Li sites from the cathodes with high Ni content are partially occupied by Ni ions due to their similar ionic radius. However, Li diffusion during the charging-discharging process is hindered by the Ni ions in the Li sites, so the high degree of cation mixing deteriorates the electrochemical performance of the cathode. The increased I(003)/I(104) values from the PB surface modification using Ta means that the Ta ions in the structure lessen cation mixing, which may enhance Li diffusion and improve the electrochemical performance of the cathodes.

**Table 1:** The cell parameters derived from Rietveld refinement and I(003)/I(104) values.

Samples	Rwp (%)	GOF	A (Å)	C (Å)	V (Å^3^)	c/a	I_003_/I_104_
Pristine	2.48	1.12305	2.8647	14.206	100.96	4.959	1.866
PB modified using Ta	2.87	1.10853	2.8610	14.202	100.67	4.964	1.972
PB modified using W	2.61	1.22256	2.8649	14.207	100.98	4.959	1.934

The PB surface modified powder using W also increased the I(003)/I(104) values compared to those of the pristine powder. As a result, the doping effect of the W ions seemed to reduce the degree of cation mixing in the LiNi_0.6_Co_0.2_Mn_0.2_O_2_ structure. The lattice parameters and unit cell volume (V) of the PB surface modified powder using W changed somewhat as well, inferring that the W ions migrated into the LiNi_0.6_Co_0.2_Mn_0.2_O_2_ and somewhat influence the bulk structure. However, the effect of W ions was not critical compared to that of the Ta ions. The PB surface modification using Ta appears to have a greater impact on the structure of the LiNi_0.6_Co_0.2_Mn_0.2_O_2_ cathode. It should be noted that the phases derived from the Ta and W sources, such as LiTaO_3_ and LiWO_3_ (or Li_2_WO_4_), which are expected to form on the surface layer, were not observed in the XRD patterns of the PB surface modified powders, possibly because of their small amount.

From the XRD and STEM-EDS analysis, it is confirmed that the Ta and W ions act as dopants of the LiNi_0.6_Co_0.2_Mn_0.2_O_2_ cathode. Due to the high heating temperature, the possibility that most of the Ta and W ions diffused inside the cathode without forming the surface coating layer cannot be excluded. To identify the existence of the coating layer, the surface of the samples was analyzed using STEM images. In general, cathodes with a high Ni content have a surface layer composed of lithium residues, so the powders were washed with water before STEM analysis to remove the lithium residues and observe the real surface layer. As shown in Supplementary Fig. S2a, the pristine powder did not show a special surface layer. In contrast, the surface of the PB surface modified powder was covered with a thin film 2–4 nm in thickness (Supplementary Fig. S2b, S2c), which is considered to be a Li–Ta–O or Li–W–O coating layer.

**Figure 5:**
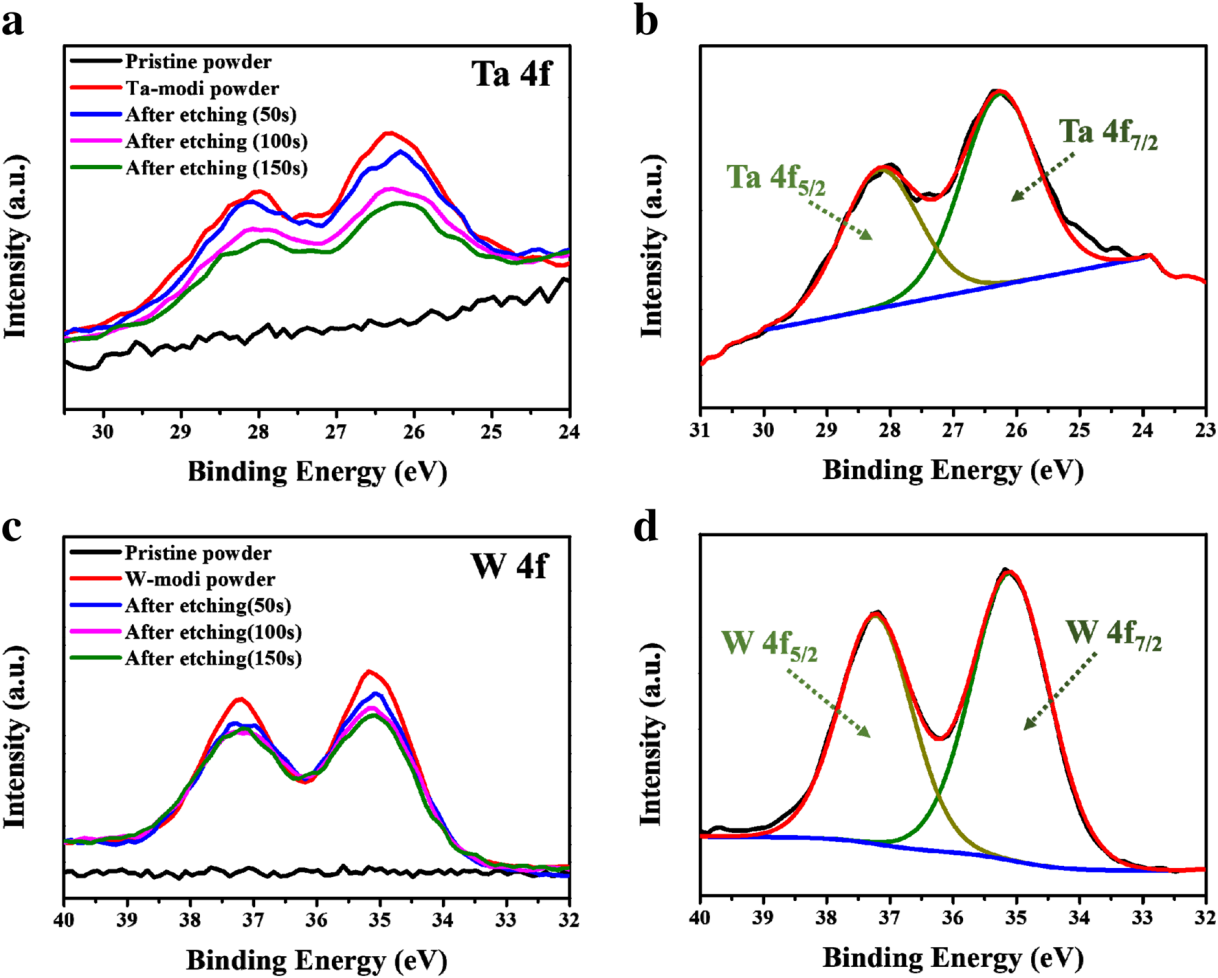
XPS spectra of the pristine and PB surface modified powders before and after etching (**a**,**b**) Ta 4f; (**c**,**d**) W 4f.

As another approach for checking the surface layer, the XPS spectra of the samples was obtained from the surface to some depth by an etching process for 50, 100, and 150 s using an ion gun. As shown in Fig. 5a, no peak was detected in the XPS spectrum of the pristine powder, whereas the PB surface modified powder using Ta clearly presented two peaks corresponding to Ta 4f_5/2_ and 4f_7/2_, as shown in Fig. 5b. Notably, the intensity of the peaks decreased with increased etching time. This means that the Ta concentration decreased as the depth increased. As shown in Fig. 5c and d, the intensity of the peaks related to the W 4f_5/2_ and 4f_7/2_ also decreased with increased etching time. Considering these results, it is clear that the Ta and W concentration at the surface is higher than at the inside the cathode, confirming the formation of a surface coating layer or at least of a concentration gradient of Ta or W. Although it was difficult to determine the exact composition of the surface coating layer, the Ta and W ions at the surface may react with Li and O and form binary oxides such as Li–Ta–O or Li–W–O on the surface of the cathode powder. This surface layer is expected to act as a protective layer against the side reactions between the cathode and sulfide electrolyte.

## Electrochemical performance of composite electrodes

**Figure 6:**
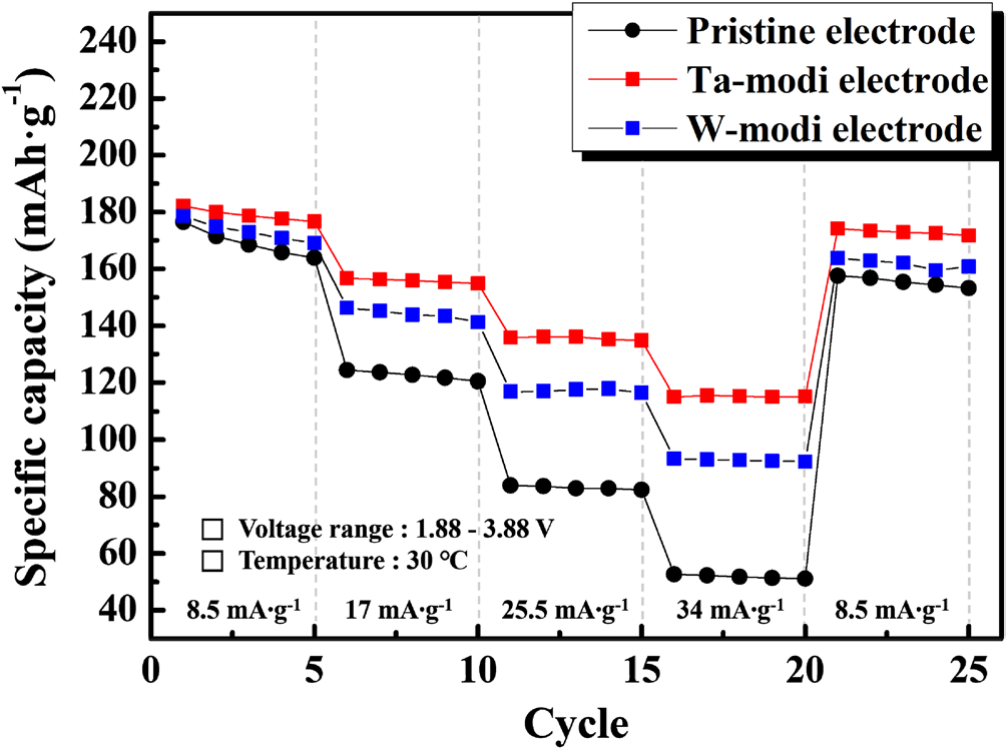
Discharge capacities of the pristine and PB surface modified electrodes at current densities of 8.5, 17, 25.5, and 34 mA·g^−1^ over the voltage range of 3.88–1.88 V.

Composite electrodes containing pristine and PB surface modified powders were prepared using sulfide solid electrolytes. They were named ‘pristine electrode’, ‘Ta-modi electrode’, and ‘W-modi electrode’, and their electrochemical properties were measured using all-solid-state cells. The voltage range of the measurement was 3.88–1.88 V, considering the voltage drop of the anode (a Li–In composite). Figure 6 presents the discharge capacities of the composite electrodes in all-solid-state cells at current densities of 8.5, 17, 25.5, and 34 mA·g^−1^. The discharge capacity of the electrodes measured using the solid electrolytes was ~ 180 mAh·g^−1^ at low current densities (8.5 mA·g^−1^). However, as the current densities increased, the discharge capacity of the all-solid-state cells distinctly reduced. At a current density of 34 mA·g^−1^, the discharge capacity of the pristine electrode was only 52.7 mAh·g^−1^. Considering that 34 mA·g^−1^ is just a ~ 0.2 C rate, and the capacity reduction in general lithium ion cells is not severe under that condition, the rate capability of all-solid-state cells is significantly inferior to general lithium ion cells. This poor rate capability of the all-solid-state cells is largely attributed to the high interfacial resistance between the electrolytes and cathodes. The side reactions and formation of the space charge layer at the sulfide electrolyte/cathode interface disturbs the movement of lithium ions and electrons, which increases the interfacial resistance.

Surface modification seems to be effective at enhancing the electrochemical performance of all-solid-state cells. As shown in Fig. 6, the PB surface modified electrodes presented a significantly enhanced rate capability compared to that of the pristine electrode. In particular, when Ta was used for modification, the discharge capacity at high current densities dramatically increased over that of the pristine electrode. Supplementary Figure S3a–d shows the charge–discharge profiles of the pristine and PB surface modified electrodes. The voltage profile and capacity at a current density of 8.5 mA·g^−1^ (initial cycle in Fig. 6) was not critically changed by the PB surface modification, as shown in Supplementary Fig. S3a and S3c. However, at high current densities (34 mA·g^−1^, 16th cycle in Fig. 6), the PB surface modified electrode presented a greatly increased capacity compared to that of the pristine electrode, as shown in Supplementary Fig. S3b and S3d. The discharge capacity and the columbic efficiency (η) of the pristine electrode were just 52.7 mAh·g^−1^ and 87.4%, respectively. The retained capacity at 34 mA·g^−1^ compared to that at 8.5 mA·g^−1^ (called capacity retention) was just 29.9%. In contrast, the capacities of the Ta-modi and W-modi electrodes were 115 and 93.3 mAh·g^−1^, respectively. In addition, the capacity retention increased to 63.2% (Ta-modi electrode) and 52.2% (W-modi electrode), indicating an enhanced rate capability through PB surface modification. Moreover, Ta and W-modi electrodes showed a significantly improved columbic efficiency (η) at 34 mA·g^−1^. The discharge capacity, capacity retention, and columbic efficiency (η) of the pristine and PB surface modified electrodes are summarized in Table 2.

**Table 2:** Electrochemical properties of pristine and PB surface modified electrodes at different current densities.

Current density	Pristine electrode		Ta-modi electrode		W-modi electrode	
	Discharge capacity (mAh·g^−1^)	Capacity^a^ retention (%)	Discharge capacity (mAh·g^-1^)	Capacity retention (%)	Discharge capacity (mAh·g^−1^)	Capacity retention (%)
8.5 (mAh·g^-1^)	176.5	100 (η* = 67.1)	182.1	100 (η = 66.9)	178.55	100 (η = 64.4)
17 (mAh·g^-1^)	124.4	70.5 (η = 90.4)	156.7	86.1 (η = 97.0)	146.3	81.9 (η = 95.2)
34 (mAh·g^-1^)	52.7	29.9 (η = 87.4)	115.0	63.2 (η = 95.9)	93.3	52.2 (η = 92.6)

η* = columbic efficiency (%).

^a^The capacity retention refers to the percentage of retained capacity at each current density compared to that at 8.5 mA·g^−1^.

**Figure 7:**
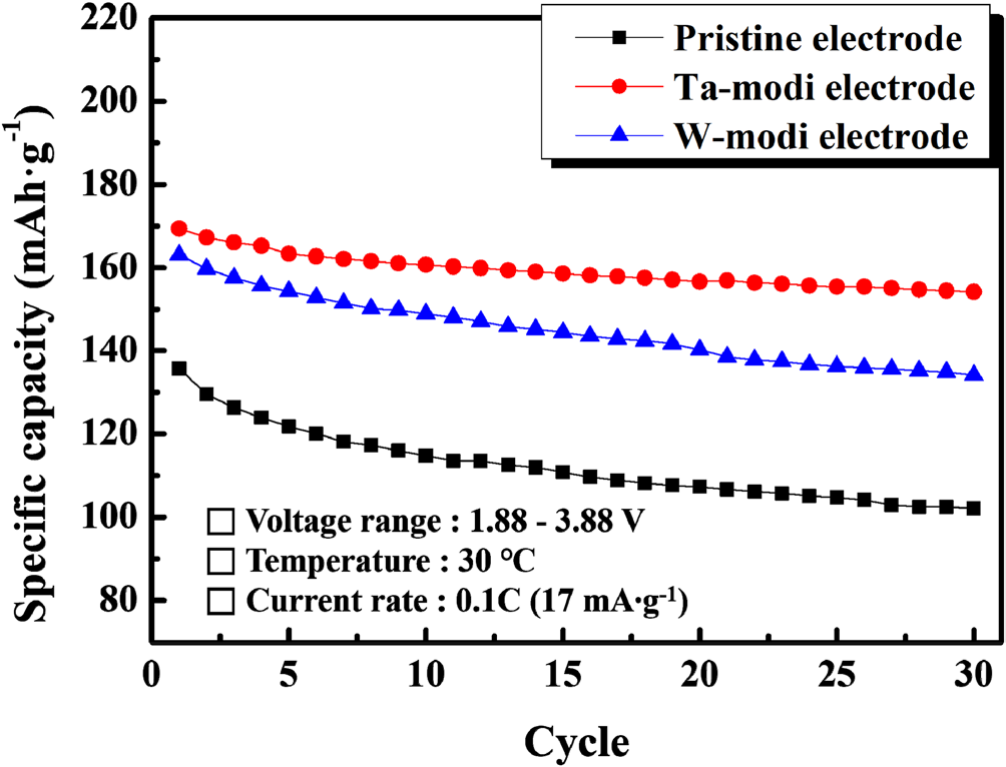
Cyclic performance of the pristine and PB surface modified electrodes at a current density of 17 mA·g^−1^.

Figure 7 shows the cyclic performances of the pristine and PB surface modified electrodes at a current density of 17 mA·g^−1^. The discharge capacity of the pristine electrodes gradually decreased over 30 cycles. After 30 cycles, the retained capacity was ~ 102 mAh·g^−1^, which is just 75% of that of the initial cycle (~ 136 mAh·g^−1^). In contrast, the PB surface modified electrodes presented a much higher discharge capacity and improved cyclic performance. The initial discharge capacities of the initial capacity Ta and W-modi electrodes were ~ 169 and ~ 163 mAh·g^−1^, respectively. The capacity retentions over 30 cycles compared to those of the initial cycle were 91% (Ta-modi electrode) and 82% (W-modi electrode).

Based on the results shown in Figs. 6 and 7, PB surface modification using Ta and W successfully enhanced the capacity, rate capability, and cyclic performance of the LiNi_0.6_Co_0.2_Mn_0.2_O_2_ cathode measured using sulfide-based all-solid-state cells. This is attributed to the synergic effect of the coating and doping derived from Ta and W. The undesirable side reaction between sulfides and the oxide cathode can be suppressed by the stable surface coating layer formed from the Ta and W sources. The doping effect could also contribute the improved electrochemical performance of cells. In particular, Ta doping significantly reduced cation mixing, which results in enhanced rate capability. Furthermore, the high Ta-O dissociation bond energy improves the structural stability of the LiNi_0.6_Co_0.2_Mn_0.2_O_2_ cathode^41^, which may reduce its reactivity with the sulfide electrolyte and stabilize the cyclic performance of the cells. The superior electrochemical performance of the Ta-modi electrode is closely associated with the fact that the structure of the LiNi_0.6_Co_0.2_Mn_0.2_O_2_ cathode was influenced by Ta doping, as shown in Fig. 4.

Although the doping effect was somewhat smaller than that of the Ta, the modification using W was effective at reducing the cation mixing as well. It has also been reported that W doping reduces the structural stress related with the phase transition that causes abrupt structural distortion^42^. This is beneficial in improving the structural stability, which leads to enhanced cyclic performance of the cathode.

Supplementary Figure S4 shows Nyquist plots of the pristine and PB surface modified electrodes in all-solid-state cells. The Nyquist plots of the cells seemed to consist of several overlapped semicircles, implying the existence of multiple resistance factors attributed to the interfacial layers and contact instability. As shown in Supplementary Fig. S4a, the original size of the semicircles before cycles increased after PB surface modification, indicating an increase in the impedance values of the cells. The surface layer formed from Li–Ta(W)–O may act as a new resistance element. However, the PB surface modification dramatically reduced the impedance value after a few cycles. The semicircle of the pristine electrode significantly increased after five cycles, as shown in Supplementary Fig. S4b. In contrast, those of the Ta and W-modi electrodes after five cycles were much smaller than that of the pristine electrode. This shows that the surface layers (such as Li–Ta–O and Li–W–O) of the cathode effectively suppress the interfacial reaction with the sulfide electrolyte during cycling.

**Figure 8:**
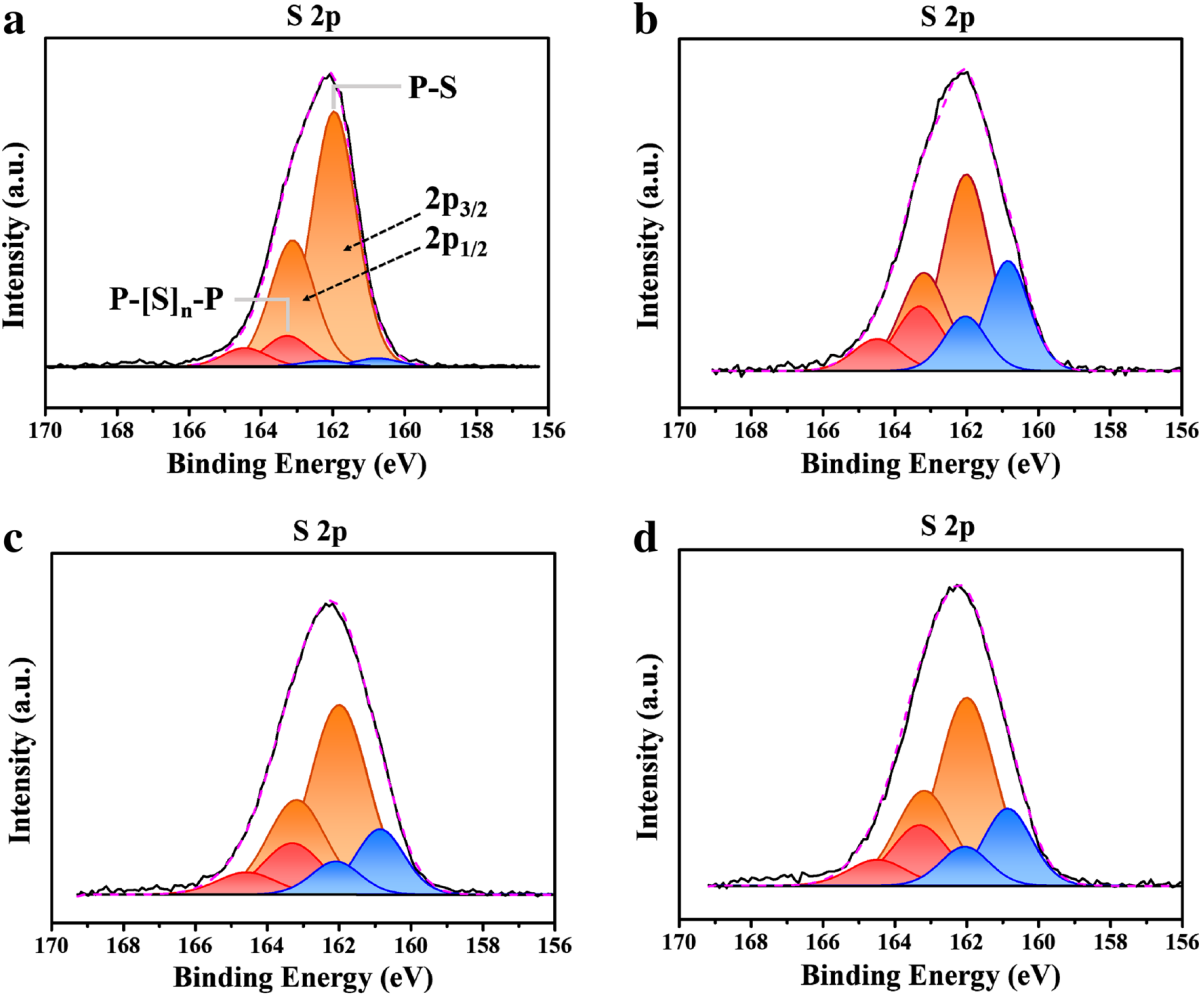
S 2p XPS spectra of the pristine electrolyte and composite electrodes of the all-solid-state cells (**a**) pristine electrode before test, and (**b**) pristine, (**c**) Ta-modi, (**d**) W-modi electrodes after 20 cycles.

To check the reaction products formed by undesirable side reactions, the pristine and PB surface modified electrodes were analyzed using XPS. Figure 8a shows the XPS spectra of the pristine electrode before cycling. The two main peaks at ~ 161.9 eV and ~ 163.1 eV (marked in orange) are related to the S 2p_3/2_ and 2p_1/2_ components of the non-bridging sulphur (S^−^) in the sulfide electrolyte^43^. The peaks marked in red are attributed to a P–[S]_n_–P type bond as in the P_2_S_7_^4−^ units^44^. The blue small peaks are mainly associated with the reaction products derived from the side reactions concerning sulfide. For XPS analysis of the composite electrode after the cycles, electrodes were collected from the all-solid-state cells that had been subjected to 20 cycles. As shown in Fig. 8b, the pristine electrodes presented somewhat different XPS profiles after 20 cycles. While the intensity of the two main peaks at ~ 161.9 eV and ~ 163.1 eV (marked in orange) decreased, the intensity of the other peaks related to the side reactions increased. Notably, the peaks at ~ 160.9 eV and ~ 162.1 eV (marked in blue) increased noticeably, showing the significant side reactions between the oxide cathode and sulfide electrolyte. The increased intensity of the peaks at ~ 163.3 eV and ~ 164.5 eV (marked in red) may be due to side reactions such as the oxidation of sulfides as well. However, PB surface modification considerably reduced the intensity of the peaks related to the side reactions. As shown in Fig. 8c and d, the XPS spectra of the Ta and W-modi electrodes (after 20 cycles) presented somewhat decreased peaks attributed to the side reactions (marked in red and blue). The enlarged XPS spectra of the composite electrodes after 20 cycles was presented in S5 to clearly compare the peaks reated to the side reactions. This XPS result confirms that PB surface modification using Ta and W was effective at suppressing the undesirable side reactions between the cathode and the sulfide electrolyte. It is inferred that the coating and doping effect of the PB surface modification results in LiNi_0.6_Co_0.2_Mn_0.2_O_2_ cathodes that are less reactive to the sulfide electrolytes. The improved electrochemical performance of the PB surface modified electrodes such as a high capacity, superior rate capability, and improved cyclic performance are associated with the effective protection effect of the PB surface modification using Ta and W.

## Summary

In this study, PB surface modification was introduced to suppress the undesirable interfacial reaction between the sulfide electrolytes (75Li_2_S–22P_2_S_5_–3Li_2_SO_4_) and cathodes (LiNi_0.6_Co_0.2_Mn_0.2_O_2_). The source materials (Ta or W) coated on the surface of the precursor diffused into the cathode and incorporated into the structure during the heating process. The Ta and W in the cathode were expected to act as dopants to improve the structural stability. From the TEM and XPS analysis, the formation of a thin coating layer that can reduce the reactivity between the cathode and sulfide electrolyte is also confirmed.

**Figure 9:**
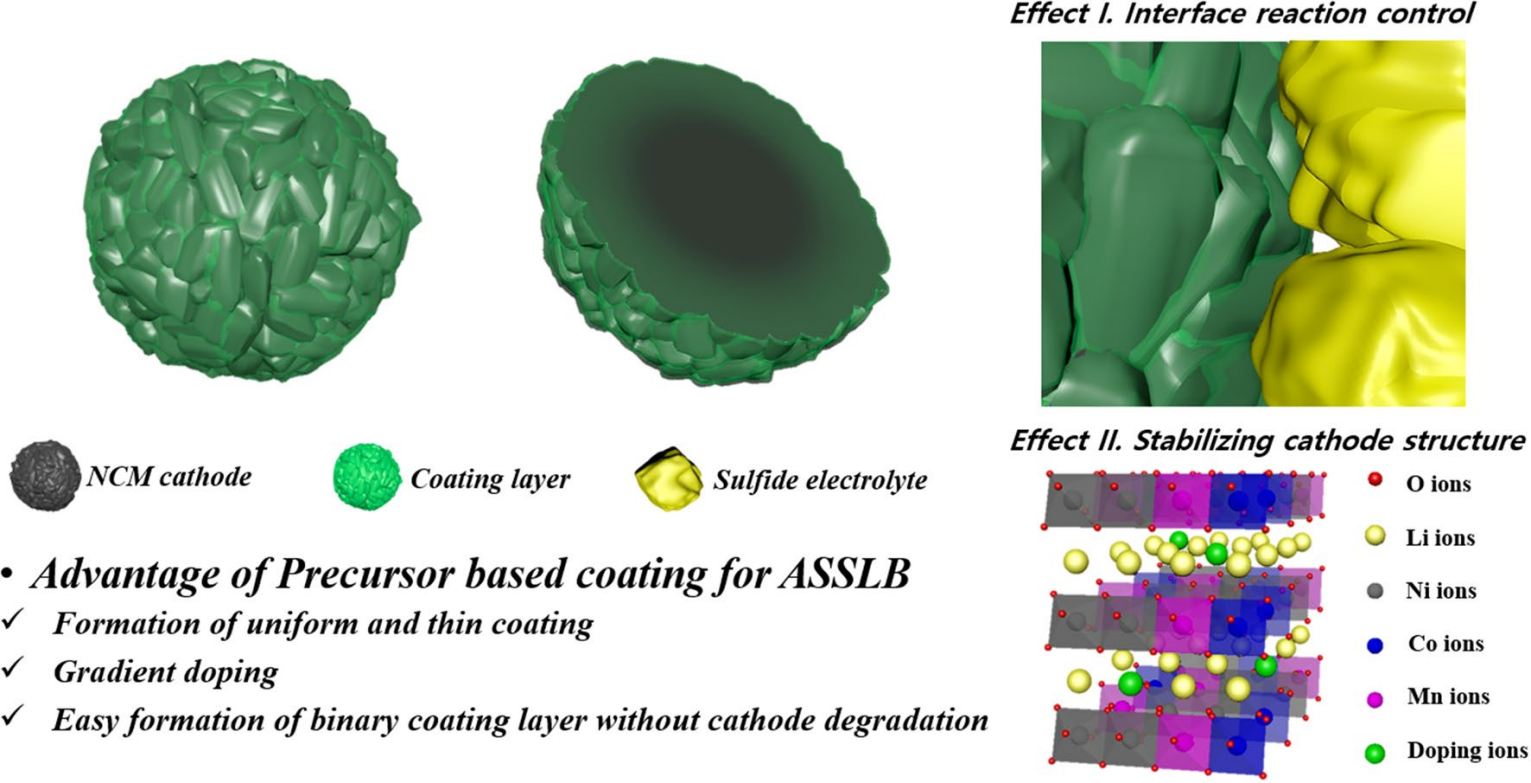
Schematic illustration the effect of precursor-based surface modification.

The PB surface modification enhanced the capacity, rate capability, and cyclic performance of the LiNi_0.6_Co_0.2_Mn_0.2_O_2_ cathode. In particular, the cathode modified using Ta showed superior electrochemical properties to those of the cathode modified using W. The PB surface modification increased the impedance value of the cells before cycling. However, it dramatically decreased the impedance value after cycling, indicating that the modification successfully reduced the interfacial resistance of the all-solid-state cells during cycling. From the XPS analysis, it was also confirmed that the PB surface modification effectively decreased the undesirable interfacial reactions between cathodes and sulfide electrolytes, which contributed to the improved electrochemical performance of the PB surface modified electrodes. It is believed that the enhanced structural stability stemming from the doping effect is synergistic with the protection effect of the surface coating layer to reduce the reactivity of the cathodes with the sulfide electrolytes. Figure 9 summarizes the effect of PB surface modification using Ta and W for cathodes of ASSBs.

## Methods

### Materials and coating procedure

The precursor of Ni_0.6_Co_0.2_Mn_0.2_(OH)_2_ was supplied by the Eco & Dream Company. To prepare the coating solution, Tantalum(V) ethoxide (Ta(OC_2_H_5_)_5_, Aldrich) and ammonium metatungstate hydrate ((NH_4_)_6_H_2_W_12_O_40_∙xH_2_O, Aldrich) were separately dissolved in anhydrous ethanol (99.9%, Aldrich) at 80 °C. The amount of coating materials was adjusted to 1 wt% based on the transition metals in the precursor. Then, cathode precursor powder was added to the coating solution and stirred at 80 °C until the solvent was completely evaporated. The dried materials were mixed with LiOH∙H_2_O at a molar ratio of 1:1.08, and prepared mixtures were heated at 500 °C for 5 h and then calcined at 800 °C (heating rate = 2 °C/min) for 10 h under an oxygen atmosphere to obtain PB surface modified LiNi_0.6_Co_0.2_Mn_0.2_O_2_ powder. For comparison, pristine LiNi_0.6_Co_0.2_Mn_0.2_O_2_ without modification was prepared using the same precursor and lithium salt.

### Sample characterization

X-ray diffraction (XRD) patterns of the pristine and PB surface modified powders were obtained using an X-ray diffractometer (Bruker D8) over the 2θ range of 5°–120° with monochromatized Cu Kα radiation (λ = 1.5406 Å). Highscore software was used to refine the lattice parameters for the Rietveld analysis. The surface morphology of the pristine and modified powder was observed using field-emission scanning electron microscopy (FE-SEM, Nova Nano 200) and scanning transmission electron microscopy (Analytic STEM I, JEOL JEM-2100F). To obtain the cross-sectional images, the powders were treated using focused ion beam (FIB) milling (Quanta 3D FEG). Then STEM and energy dispersive X-ray spectroscopy (EDS) were employed to investigate the element diffusion inside the cathodes. X-ray photoelectron spectroscopy (XPS, Thermo Scientific K-Alpha plus) was used to confirm the surface coating layer. The powder was etched using an ion gun to obtain the depth profile of the XPS spectra.

### All-solid-state cell fabrication

For electrochemical testing, all-solid-state cells were fabricated using sulfide solid electrolytes (Jeong Kwan Co., LTD, 75Li_2_S–22P_2_S_5_–3Li_2_SO_4_) according to the previously reported method^3,31^. The cathode mixture for the composite electrode was prepared by mixing the cathodes (pristine or PB surface modified powders), sulfide solid electrolytes, and carbon black (Super P) at a weight ratio of 70: 30: 2. To form a solid electrolyte layer as a separator, 0.2 g of the sulfide electrolyte was compressed under 30 MPa pressure in a Φ16 mould. Thereafter, the composite electrode (cathode) layer was formed on one side of the solid electrolyte using 0.02 g of the cathode mixture and carbon-nanotube paper (Hanwha Chemical). The anode electrode layer was formed on the opposite side with 0.05 g of Li–In powder and nickel foil. Each compression process was performed at 30 MPa. The cathode/electrolyte/anode assembly was placed inside a 2032 coin-type cell.

### Electrochemical properties

The electrochemical properties were measured with reference to the reported works^3,31^. The cells were subjected to galvanostatic cycling (WonATech voltammetry system) over a voltage range of 3.88–1.88 V at various charge–discharge rates. XPS (Thermo Scientific K-Alpha plus) was employed to analyse the reaction products on the composite electrodes containing pristine and PB surface modified samples. The all-solid-state cells were cycled 30 times, then the composite electrodes were separated from the cells and stored in a dry box. The electrodes were held under vacuum transfer while being transferred to the instruments and etched to ~ 100 nm using Ar sputtering to remove contamination on the surface before analysis.

## Supplementary information

Supplementary file1 (PDF 668 kb)
