# Supplementary material for: Precursor-based surface modification of cathodes using Ta and W for sulfide-based all-solid-state batteries
Source: Sci Rep. 2020 Jun 29;10:10501. doi: 10.1038/s41598-020-67493-6 (PMC7324361; doi:10.1038/s41598-020-67493-6)
Supplement: Supplementary file 1 — Supplementary file1 (PDF 668 kb) [file 41598_2020_67493_MOESM1_ESM.pdf]

Precursor-based surface modification of cathodes using Ta and W for sulfide-based all-solid-state batteries

Chung Bum Lim and Yong Joon Park*

Department of Advanced Materials Engineering, Kyonggi University, 154-42, Gwanggyosan-Ro, Yeongtong-Gu, Suwon-Si, Gyeonggi-Do, 16227, Republic of Korea

*Corresponding author

Ph: +82-31-249-9769; E-mail: yjpark2006@kyonggi.ac.kr

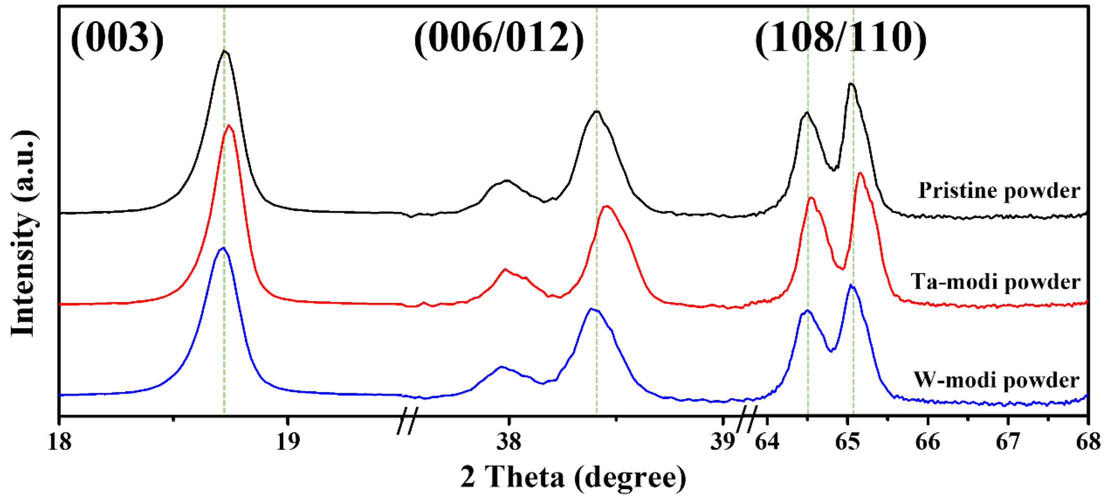

Supporting S1 | XRD patterns magnified near (003), (006)/(012) and (108)/(110) peaks.

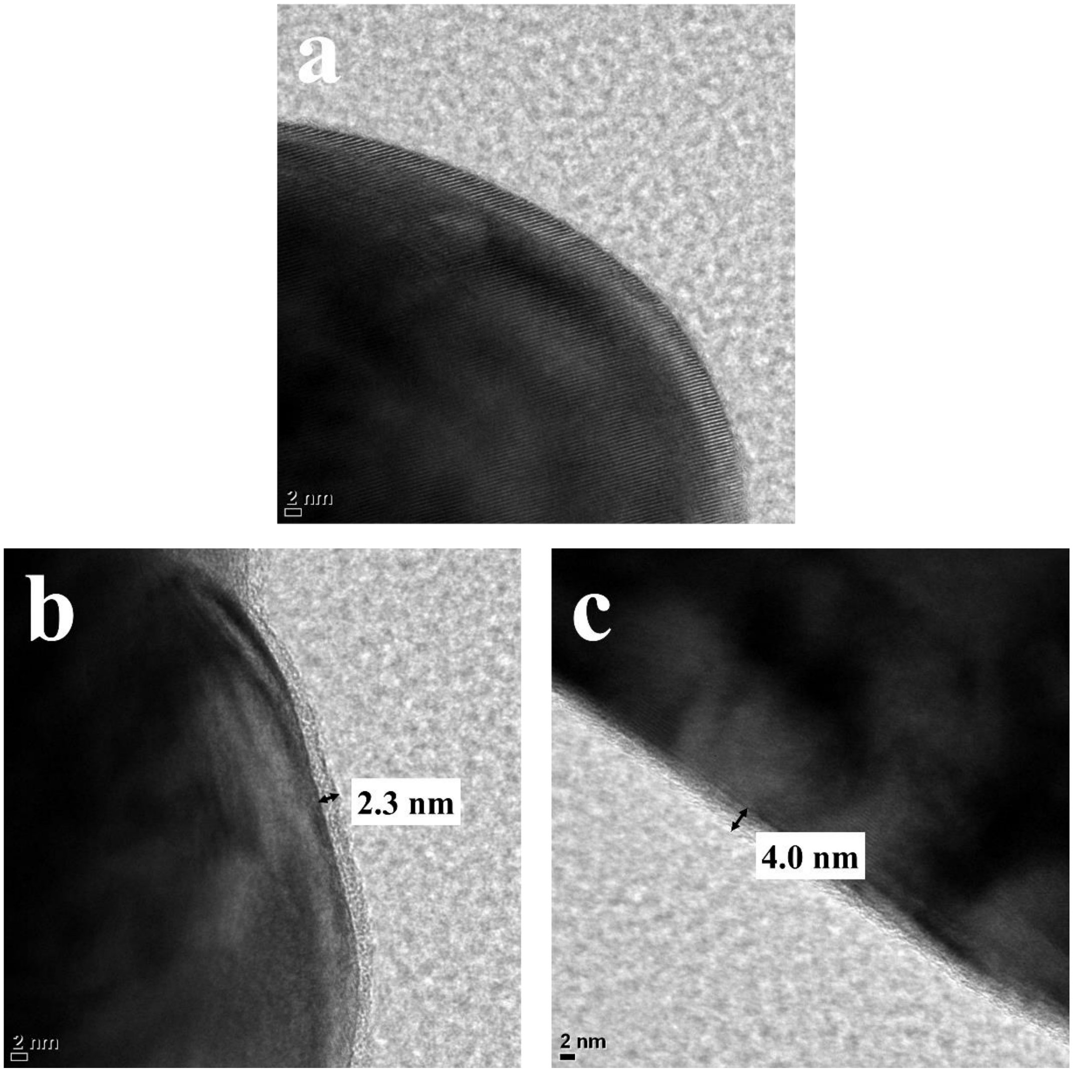

Supporting S2 | TEM images focused on the surface of the samples (a) pristine powder, (b) PB surface modified powder using Ta, and (c) PB surface modified powder using W

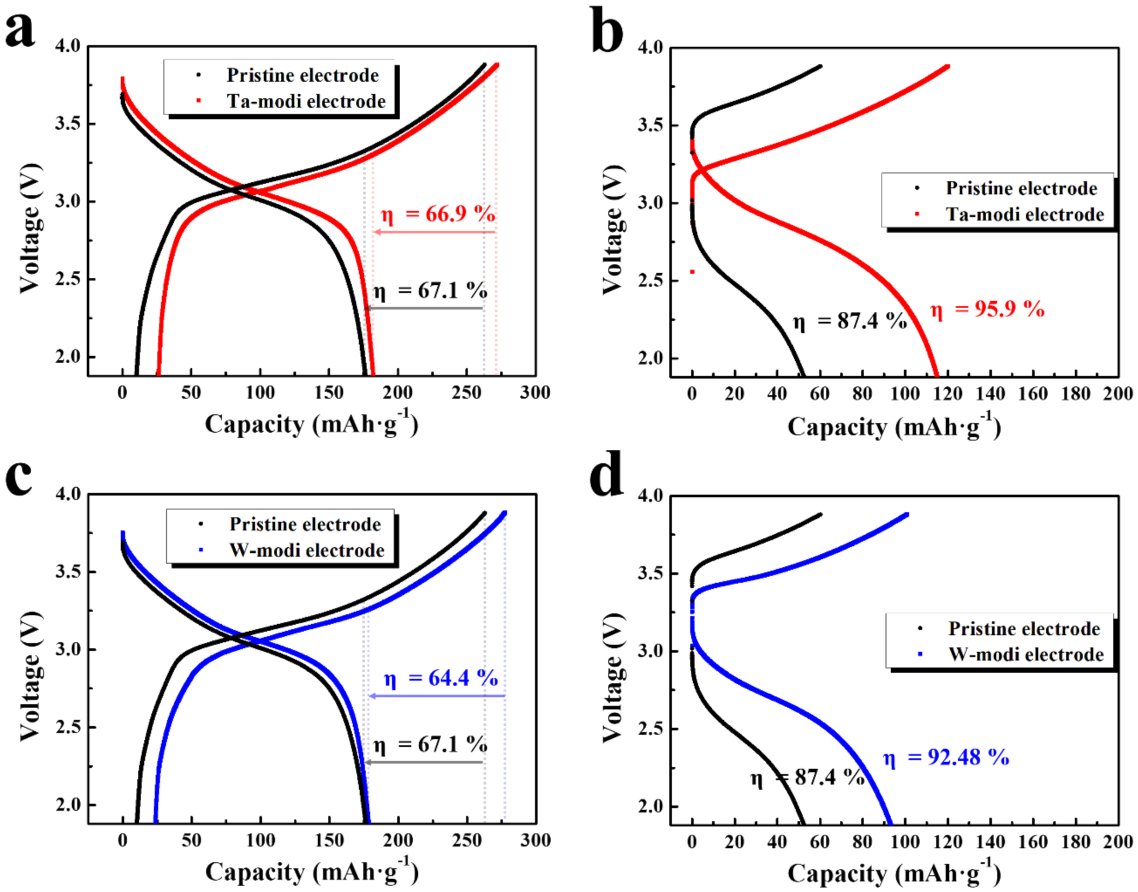

Supporting S3 | Charge-discharge profiles of the pristine and Ta -modi electrodes at (a) 8.5 and (b) 34 mA·g^{-1}, those of pristine and W-modi electrodes at (c) 8.5 and (d) 34 mA·g^{-1}

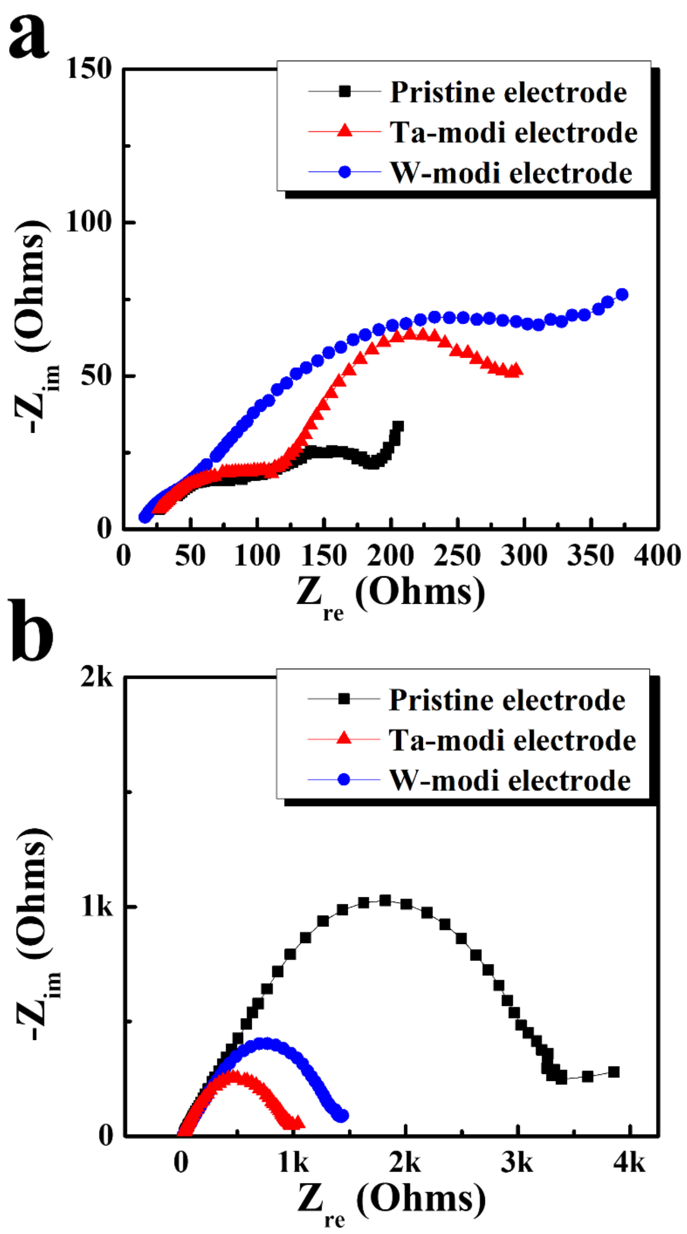

Supporting S4 | Nyquist plots of the pristine and PB surface modified electrodes in all-solid-state cells (a) Before cycles, (b) after five cycles in a charged state

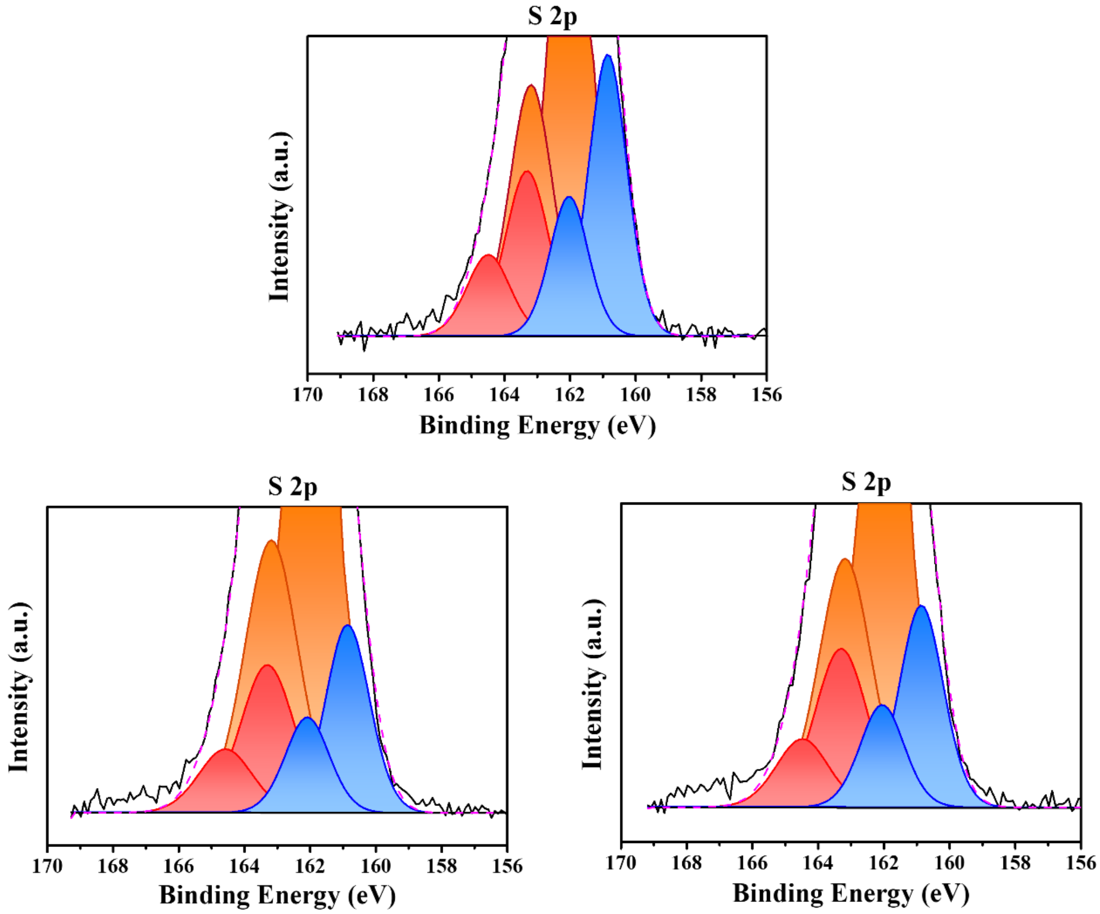

Supporting S5 | Enlarged S 2p XPS spectra of the composite electrodes after 20 cycles (a) pristine, (b) Ta-modi, (c) W-modi electrodes
